# Cryptographically Secure PseudoRandom Bit Generator for Wearable Technology

**DOI:** 10.3390/e25070976

**Published:** 2023-06-25

**Authors:** Michał Melosik, Mariusz Galan, Mariusz Naumowicz, Piotr Tylczyński, Scott Koziol

**Affiliations:** 1 Department of Computer Science and Telecommunications, Poznan University of Technology, Piotrowo 3A, 60-965 Poznan, Poland; 2 Intel Technology Poland, Juliusza Słowackiego 173, 80-298 Gdańsk, Poland; 3 Electrical & Computer Engineering Department, Baylor University, One Bear Place #97356, Waco, TX 76798, USA

**Keywords:** random number generator, wearable, FPGA, Cryptographically Secure Pseudo-Random Bit Generator, seed generator, wearable entropy source, entropy source

## Abstract

This paper presents a prototype wearable Cryptographically Secure PseudoRandom Bit Generator CSPRBG (wearable CSPRBG). A vest prototype has been fabricated to which an evaluation board with a ZYBO (ZYnq BOard) Zynq Z-7010 has been mounted using tailoring technology. In this system, a seed generator and block cryptographic algorithms responsible for the generation of pseudo-random values were implemented. A microphone and an accelerometer recorded sound and acceleration during the use of the prototype vest, and these recordings were passed to the seed generator and used as entropy sources. Hardware implementations were made for several selected Block Cryptographic algorithms such as Advanced Encryption Standard (AES), Twofish and 3DES. The random binary values generated by the wearable CSPRBG were analyzed by National Institute of Standards and Technology (NIST) statistical tests as well as ENT tests to evaluate their randomness, depending on the configuration of the entropy sources used. The idea of possible development of the wearable CSPRBG as a System on Chip (SoC) solution is also presented.

## 1. Introduction

In the research on most types of hardware random generators, the possibility of using wearable technology to create secure sources of entropy is disregarded. The security of such entropy sources is critical because they generate the values used to initialize pseudo-random algorithms. This paper presents a concept of providing the best diversified entropy source possible for mobile and wearable microelectronic systems. The quality and level of randomness has an impact on the security of the cryptographic key generation process [1]. Typically, studies on how random generators create random sequences based on entropy sources concern theoretical or software considerations without addressing the specifics of the hardware layer [2,3]. In our paper, we focus on the design of the hardware layer for wearable technology. Sources of entropy may fail—e.g., external sensors may be damaged or exposed to special attacks. The aim of the attacks may be to influence the observed phenomenon monitored by a given sensor or to influence the way the sensors measure the phenomenon. In addition, the use of only one type of sensor may cause a risk. For example, if the phenomenon registered by the singular sensor type does not show changes, then the values resulting from such measurements will be characterized by little or no dynamics of change. The development of wearable technology shows that the processing of sensitive data requiring cryptographic security is becoming increasingly common [4,5,6,7,8]. This implies the need for the development of cryptographic modules adapted to the specifics of wearable technology.

It is well known that random number generators are one of the fundamental components of security systems. The process of their design and their structure has been well standardized. The National Institute of Standards and Technology (NIST), for example, provides recommendations for designing deterministic random number generators [9]. Using the same value repeatedly in the process of initializing pseudo-random algorithms raises risks for attacks that affect DRBG (Deterministic Random Bit Generator) initialization [9]. Despite the knowledge of these standards, it is still challenging to provide multiple efficient and attack-resistant entropy sources in the design process of random generators [10,11,12,13,14,15,16,17,18].

*The aim of this paper is* to present the concept of a prototype of wearable Cryptographically Secure Pseudo-Random Bit Generator (wearable CSPRBG). This prototype takes into account the specifics of wearable technology—its final use in environments with changing parameters such as the user’s location, speed of movement, ambient temperature and sound recorded from the environment. The security of the process of forming random bitstreams of the wearable random generator will be guaranteed by the use of a block cryptographic algorithm. The algorithm is initialized with a vector of bits that are first formed by mixing physical values recorded by a set of sensors of the prototype wearable vest and then transmitted to the seed generator.

**Figure 1:**
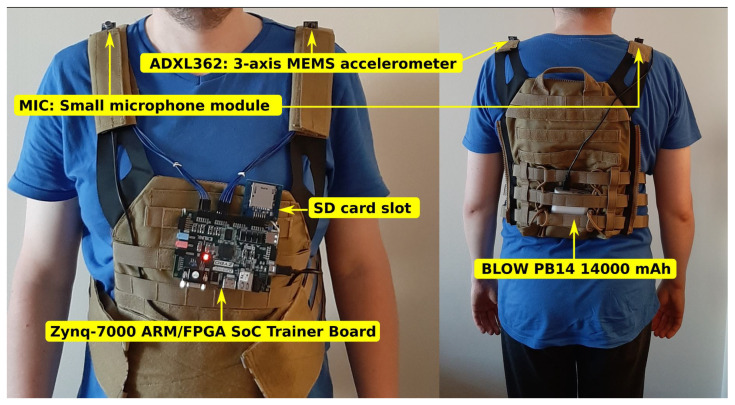
Prototype wearable-CSPRBG before starting programming. An FPGA circuit board was mounted below the chest. Two independent entropy sources were mounted on the arms—a microphone to record ambient sound changes and an accelerometer to record acceleration. A power module was mounted on the back.

Figure 1 shows a prototype of a wearable generator based on a Field Programmable Gate Array (FPGA) circuit board. A modular vest was chosen as the base for the wearable technology, which allowed the ZYBO board to be mounted under the chest using seams. A set of sensors was mounted on the arm of the vest. The readout from the sensors is the source for creating seed values prepared by the seed generator (in the FPGA device) to initialize the deterministic algorithm (also in the FPGA device). The modular structure of the vest allowed for mounting a power bank of type Power Bank BLOW PB14 (14,000 mAh), necessary to power the entire prototype. The power bank was mounted on the user’s back.

The next two subsections describe two primary benefits of the wearable entropy generator.

### 1.1. Reducing Dependence on Externally Downloaded Entropy Sources

Our proposed prototype of a wearable vest with a random generator guarantees non-dependence on services such as Entropy as a Service (EaaS) [19] for Internet of Things (IoT), which require an additional communication layer with an external service usually in the cloud. Mobile electronic systems have led to an increasing demand for EaaS services. Such services do not generate ready-to-use random passwords per se but, provide reliable entropy for DRBGs consistent with NIST recommendations detailed in [9]. The use of EaaS requires the establishment of an additional secure Internet connection between the electronic system using the service and the remote entropy provider. Such a scenario is particularly important when it is not possible to download the entropy in advance and store it on a dedicated data device. This is a limitation of EaaS services, since in situations where it is not possible to obtain an online connection to the EaaS it may be the circumstance that the entropy previously downloaded and stored on a dedicated storage medium has already been consumed for DRBG initiation. According to NIST recommendations detailed in [9], the values used for initialization of pseudo-random generators cannot be reused. A solution to such a situation where a missing entropy occurs may be to design a system inspired by wearable technology. This would provide continuous access to new entropy that is created by sensing changing external factors and mixing them together. Such a design allows the formation of seed values for pseudo-random generators including cryptographically secure random generators designed according to NIST recommendations described in [9].

### 1.2. Reducing Vulnerability to Single Sensor Attacks

Attacks on entropy sources—and thus on seed generators—has been a significant security problem for years, and is a particularly popular topic in the context of threats from hardware Trojans [18,20]. Using multiple types of sensors minimizes the risk of attacks on selected individual sensors. In case of an attack on only one of the sensors, e.g., a sound sensor, the entropy will still be delivered to the wearable CSPRBG as a result of mixing the values coming from the motion sensor (MEMS ADXL362) and the chaotic generator (logistic map). To make it even more difficult to attack individual sensors, selected sensors may in the future be hidden inside the textile so that they go unnoticed, and access to them is made even more difficult. Our goal was to develop such a random generator that is: (1) suitable for wearable technology, and (2) can also be implemented independently by the widest possible range of electronics engineers with popular electronic equipment. Independent implementation guarantees the non-existence of Trojans, because the designer has full control over the entire process of design, implementation, and testing.

The rest of the paper is organized as follows: Section 2 describes the security of random sequence generators, Section 3 describes the hardware used to implement the wearable entropy generator, Section 4 describes performance of the wearable CSPRBG hardware and its speed of generating pseudo-random strings, Section 5 describes the randomness of the system, Section 6 describes future development and Section 7 provides a summary.

## 2. Random Generator Security

When designing random generators for cryptographic purposes, one may distinguish three basic divisions based on how the generators produce the random sequences [21]:

- True Random Bit Generators (TRBG) produce random sequences based on direct observation of a physical phenomenon.
- Pseudo-Random Bit Generators (PRBG) produce random sequences based on a deterministic algorithm initiated by an entropy source.
- Cryptographically Secure Pseudo-Random Bit Generators (CSPRBG) produce random sequences based on a strong cryptographic algorithm and a protected entropy source.

Knowledge of the deterministic algorithm used to design a Pseudo-Random Bit Generator (PRBG) and knowledge of the subsequent seed values poses the important security problem of the predictability of the random sequences generated by PRBG generators. A solution to this problem is the use of Cryptographically Secure Pseudo-Random Bit Generators (CSPRBG), in which a strong block-based cryptographic algorithm acts as one of the main modules of the entire random generator. Using a deterministic block-based cryptographic algorithm in the role of the algorithm makes it basically impossible to predict subsequent random sequences. An additional safeguard in such implementations is the use of the SHA-3 function before the seed value is used in a new random values generation cycle [22]. Among the most popular implementations of CSPRBG is the FORTUNA generator.

When the random bit generator is implemented in a classical operating system, the values initiating the operation of the generator are created from multiple sources of entropy, which are from typical processes occurring in the operating system, e.g., packet monitoring, peripheral device activity, GPU temperature, fan speed, etc. [3]. If this generator is implemented as a microelectronic device without an operating system, the designer faces a real and critical problem which is the lack or significantly limited number of entropy sources that can be directly integrated with such a device.

Considering the dynamic trend of wearable technology development, we propose a system concept that uses as entropy sources the values recorded from the wearable CSPRBG sensors. The wearable CSPRBG sensors collect readings of physical quantities during wearable CSPRBG usage. The inspiration for the implementation of digital CSPRBG was Fortune’s algorithm [23].

## 3. Hardware Layer

In the context of wearable technology, it is important to consider the choice of an appropriate hardware layer for the primary prototyping of microelectronic systems. The Application Specific Integrated Circuit (ASIC) design process is a particularly complex, time-consuming and expensive issue if only a single prototype is made [24]. Reconfigurable analog circuits called Field Programmable Analog Arrays (FPAAs) [25,26,27] and reconfigurable digital circuits called Field Programmable Gate Arrays (FPGAs), however, provide alternative methods for creating custom circuits quickly and relatively inexpensively. FPAAs have been used for stochastic computation [28]; however, this work uses digital methods. Therefore, building single FPGA-based prototypes provides an appropriate foundation for prototyping more complex wearable devices in the future. A well-developed wearable prototype can be treated as a reference model for the target ASIC, the use of which is reasonable for large-scale commercial product manufacturing. The adoption of ASICs as a base for prototyping does not allow as much flexibility for modification at the hardware layer during the design, research and test stages as does the modular approach using reprogrammable FPGAs. This is the main reason that FPGAs are being successfully used more and more in prototyping wearable solutions [29,30,31]. FPGA prototyping most often uses hardware description languages such as Very High-Speed Integrated Circuit Hardware Description Language (VHDL), or VHDL-AMS (VHDL for Analog and Mixed Signals) if the digital part being modeled requires interaction with an external analog module [32,33].

**Figure 2:**
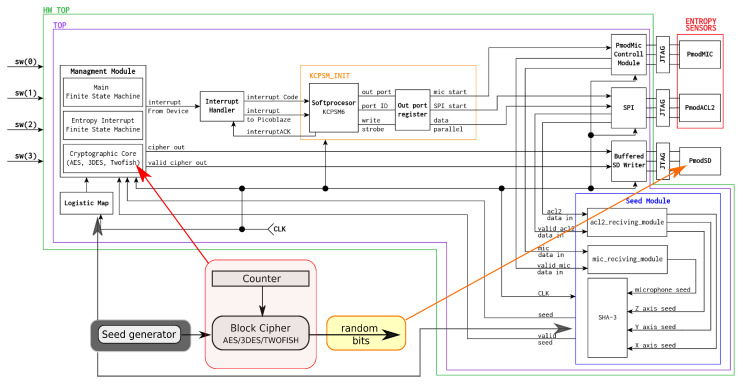
Schematic of the hardware implementation of a wearable CSPRBG using a Zynq Z-7010 and miniature microphone and ADXL362 accelerometer as two independent entropy sources. The lower left part of the figure shows the simplified CSPRBG model. The CSPRBG seed generator (blue) uses the SHA-3 algorithm to mix the values to mix the values obtained from the entropy sources and accumulated in the *ac12_reciving_module* and *mic_reciving_module*. Having completed the mixing process, the seed value is passed to management module (with the AES, 3DES, Twofish cryptographic algorithms implemented) to form a random bitstream. Hardware switch configurations (sw(0), sw(1), sw(2), and sw(3)) are described in Table 2.

Figure 2 shows a schematic of the hardware layer implemented on an FPGA board. The sources of IPCore used are shown in Table 1. A Digilent brand ZYBO (ZYnq BOard) with a seventh-generation core System on Chip (SoC) Zynq Z-7010 was chosen as the main hardware layer. However, this kit did not have the ability to collect information from the external environment such as acceleration and sound recording, so it was expanded to include three external modules to make these types of measurements. Due to the requirements of maintaining maximum flexibility and portability between different hardware platforms, the use of built-in peripheral circuits was eliminated and external modules were used.

In order to measure the sounds, the PmodMIC external module was used, which is a microphone with a 12-bit digital interface. Communication with the module is done using Serial Peripheral Interface (SPI) protocol, and the read data are transferred in 16 clock cycles. The acceleration reading is done by the PmodACL2 sensor using the MEMS ADXL362 chip that allows data recording in three axes. Communication with the chip is done via SPI protocol. In order to read the collected data from a given axis, a read command is sent, followed by the address byte, after which the system returns two data bytes: 12 bits of which carry information about the read acceleration, and the remaining four bits are redundant and result from the communication protocol used. The last external module used in the project is PmodSD which allows one to read and write data from an SD card for later evaluation of randomness of recorded bitstreams. Communication with PmodSD is performed using SPI protocol.

The choice of where to mount the sensors was dictated by the type of vest. The user makes independent movements with the shoulders while walking. By placing the sensors near the shoulders, the accelerometer will generate the greatest variety of results [41]. The choice of where to mount the sensor depends strongly on the characteristics of the wearable solution its purpose and limitations. In the presented prototype, the main limitation is the fact that the vest has no material on the arms. Thus, the greatest measurement variability is possible only in the shoulder area. Similarly, the microphone positioned highest has the ability to collect sound from the largest possible range. Placing it near the stomach or back, would limit the space from which sound is collected. With a view to further development of the prototype in the future, the possibility of integrating sensors, for example, on the wrist [42] should be considered. However, the authors of such solutions do not take into account the possibility of exposing such prototypes to failure, for example, in case of hitting the wrist against some obstacle. Mounting sensors near the shoulders for this reason seems more secure, especially in the context of early usable prototypes.

The orange rectangle in the center of Figure 2 marks the PicoBlaze embedded 8-bit Reduced Instruction Set Computer (RISC) microcontroller core. The microcontroller’s purpose is to provide communication between the wearable sensors mounted on the arms of the prototype vest and the CSPRBG hardware implementation in the ZYBO attached under the chest. On the right of Figure 2, the wearable sensors are marked in red: the microphone that records ambient sounds (PmodMIC), and the ADXL362 chip (PmodACL2) that measures acceleration. In wearable technology, a microphone captures not only ambient sounds but also the air noise produced on the material protecting the microphone membrane. If we would use a stationary device, then we risk that the sound pattern, in certain time intervals and strictly defined conditions, may be repeatable. The wearable device–and thus, the user in motion, as well as the motion itself, provides a basis for generating varying entropy. The values recorded by the sensors are directly passed to the seed generator marked in blue on Figure 2. The module marked in blue forms the seed values used to initialize the wearable CSPRBG. Note that the data passed to the seed generator is additionally secured using the SHA-3 mixing function. When the seed value is ready, it is sent to the Management Module containing selected implementations of block cryptographic algorithms and finite state machines controlling all modules. In addition, the wearable CSPRBG prototype we designed has an additional protection solution for the situation when the seed value could not be generated due to the lack of new entropy coming from the sensors. This protection is denoted in the rectangle Logistic_map in Figure 2. This is a digital implementation of the logistic map, which is a discrete chaotic system. When the seed generator cannot create a new seed value, the process of generating random values is kept by taking chaotic values from logistic_map that initiates a cryptographic deterministic algorithm. The raw data from the individual sensors are not used to create seeds, but is used in secure mixing of their values with each other and the values from the chaotic generator in the form of logistic maps.

The PmodSD block represents the external peripheral circuit that allows recording of the output binary random strings generated by the wearable CSPRBG. The green color indicates the module named as HW_top, which is implemented directly in the FPGA chip mounted under the chest of the wearable vest. The sensors marked in red are mounted on the arms of the wearable vest. Table 3 compares the FPGA hardware resource usage when three different block cryptographic algorithms are used as IPCores from Table 1. The last column in Table 3 quantifies the difference between the maximum FPGA utilization percent and the minimum FPGA utilization percent for a given FPGA resource. The results indicate that all three algorithms use approximately the same amount of FPGA resources. The outlier of resource utilization is the Block Random Access Memory (BRAM) utilization where there is a 15% difference between the 3.3% utilization of 3DES and the 18.3% utilization of AES.

## 4. Wearable CSPRBG Performance

The performance of the designed wearable CSPRBG was examined based on the generation time of 1,000,001,536 bits which corresponds to generating almost 8 million 128 bit keys and about 16 million 64 bit keys in about 4 h. Based on the above data, the effective speed of the device was calculated to be 69 Kbps. The speed of generating pseudo-random strings is due to the frequent entry of the prototype into the waiting state. This is done to collect new data from the sensors that form the entropy sources. Taking into account that the number of unique 128 bit or 256 bit keys necessary for a single device and user can be up to several dozen random values, this time is reduced to several minutes.

## 5. Randomness of Wearable CSPRBG

**Figure 3:**
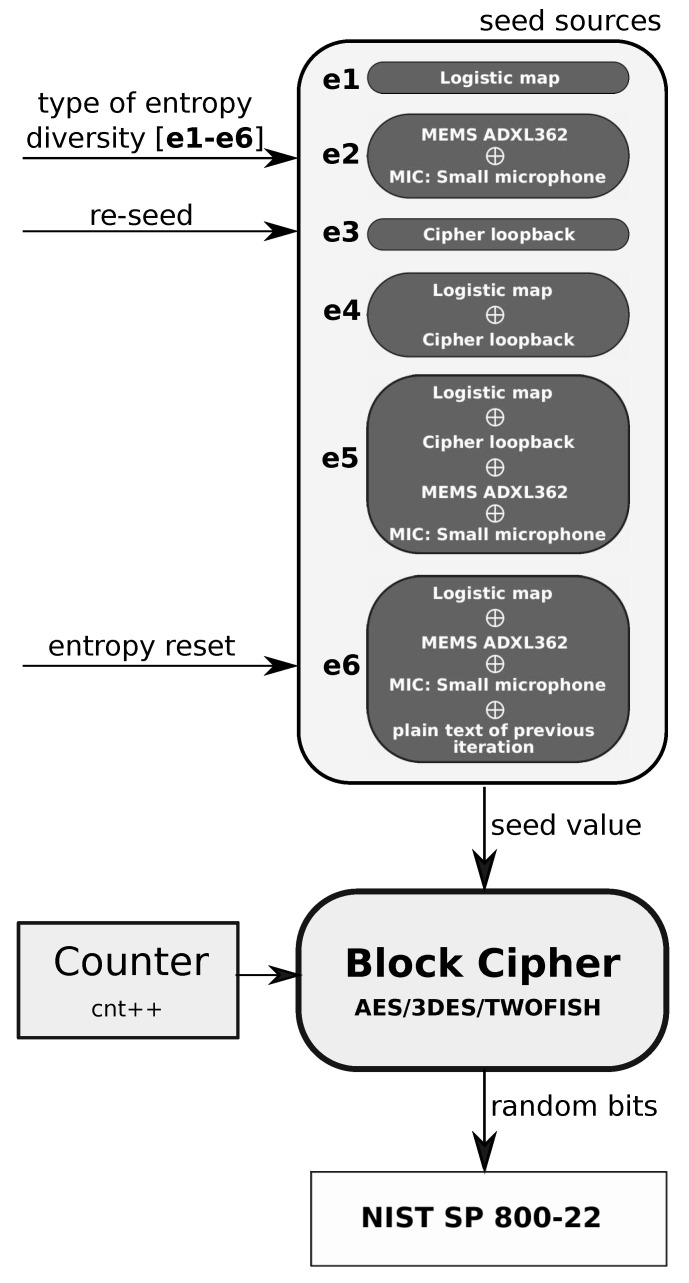
Configuration of entropy sources used in NIST and ENT test assessment.

The different configurations of entropy sources are shown in Figure 3. The randomness testing of the bit string generated by the wearable CSPRBG prototype was done for each of the e1-e6 configurations. From each of the six configurations in Figure 3, a seed value is created, and this seed value is then used to initialize the cryptographically secure random generator. From this, the currently used cryptographic algorithm (AES/3DES/TWOFISH) returns a random bit stream that is assessed by NIST tests. The system is designed so that the recorded data are used only for the purpose of creating seed values. The recorded data are not used anywhere else. To evaluate the randomness of the generated binary sequences by the wearable CSPRBG, the data were saved to an external SD card. The data thus stored with the assistance of a PC were tested with all the tests available in the NIST package with the default parameters recommended in [43]. The results show how the values obtained from individual entropy sources (or mixing of several entropy sources, Figure 3), affect the randomness of the bits obtained from each particular cryptographic algorithm. A summary of the results obtained using the NIST tests is presented in Table 4 with different configurations of entropy sources, from which values are passed to the particular cryptographic algorithm. The values presented in the summaries represent information about a passed (0) or failed (1) test. For tests that consist of sub-tests, the proportion between the sum of all tests and the number of failed tests is presented. An example of such a test is the non-overlapping test which consists of 148 independent tests, so a score of 1/148 presented in the table means that one of 148 tests failed. A detailed description and mathematical interpretation of each test can be found in [43]. From the point of view of assessing randomness (with NIST test results presented in the assumed form), it is crucial to indicate the sources of entropy for which the total number of tests shows an increase in the number of tests failed. Orange color indicates the results of statistical tests for sources of entropy in which the number of failed tests is greater than two and less than 10. Red color indicates the cases in which the negative results of statistical tests were greater than 10. The biggest problems with randomness are shown by non-overlapping tests. It was assumed that with two failed tests (out of 188 made), the binary sequence analyzed is random. A number of failed tests greater than two, due to its rigorous application for cryptography, was considered a potential indication of problems that could arise with randomness in the analyzed bit sequence.

According to the NIST recommendations on Deterministic Random Number Generators (DRNG), it is necessary to develop such generators that, from entropy sources, will be able to form sequences of bits that exhibit properties using deterministic pseudo-random algorithms [9]. In DRNG, the generation of random bits takes place only after values from entropy sources are mixed together and subjected to DRBG processing [3,44]. It should be noted that in random generators, e.g., those on Linux systems, randomness is not checked at the level of entropy sources but at the level of the final bit sequence created by the entire random generator. From a practical point of view, collecting data for randomness testing directly from entropy sources takes too much time and is not practical to do. Therefore, randomness in the statistical sense is obtained only by the actions of the DRBG algorithm, as recommended by NIST, and just on the basis of the analysis of such a bitstream, the assessment of randomness by NIST statistical tests is made [9]. For this reason, in our article, we present the results of NIST tests concerning the output sequence of the wearable CSPRBG, and not from the values formed on the observations made by individual sensors mounted on the vest.

If the generator relied on a single entropy source and, in addition, a poorly chosen or designed one, the values would have identical values each time the initiating values were created. This would constitute a forbidden situation, which NIST explicitly warns against in its recommendations [9]. This problem is well known and increasingly reported in research on secure initialization of random generators for cryptographic systems [10,11,12,13,14,15,16]. Having a well-designed PRBG is not sufficient, because in the case of the described entropy problems, random generators implemented serially on several different devices can generate almost identical random sequences. Therefore, it is important to use such entropy sources in random generators that take into account the specifics of the target destination of the random generator and the conditions under which it will operate. In the context of the presented article, this is wearable technology. In the solution presented in this article, threats of this type will not occur because, in this case, in parallel with a strong cryptographic algorithm, a diversified entropy pool has been designed. This pool is further assisted by an internal initialization mechanism based on a discrete chaotic system and using Secure Hash Algorithm (SHA) functions.

An analysis of the results in Table 4 shows that the key factor in providing randomness is primarily the specific cryptographic algorithm used. In a situation where the wearable CSPRBG prototype uses the AES algorithm, the weakening of the level of randomness of the bits returned by this prototype is not affected by any of the configurations of entropy sources from Figure 3. However, the NIST results for the AES algorithm must be confronted with the possibility of mass production of the prototype presented in this paper. If the prototype were to be serially replicated, and if only the chaotic generator (logistic map—configuration e1) were used as a source of entropy in its successive copies, there would be a threat of an identical random sequence being replicated by each of the prototypes. The sequences generated by each prototype copy will be identical if the logistic map in each prototype has the same initial condition set. Independent checking of these sequences on various independent devices by NIST testing will not detect this threat. Therefore, prevention of this type of hazard must be anticipated from the design stage of the entire prototype. This consideration shows how important it is to design a seed generator with maximally varying entropy. For this reason, the additional entropy input from the MEMS ADXL362 and the miniature MIC is intended to prevent duplication of identical seed values in successively identical physical copies of this prototype. With the default configuration of the two prototypes of the device, the analog values recorded from the external environment provide diversity in the seed values even when the user has not changed the initial condition value in the logistic map module. In the presented wearable CSPRBG prototype, such differentiation is achieved precisely through the entropy source configurations in Figure 3 (especially configurations e5 and e6).

In July 2018, 3DES was announced as a cryptographic algorithm to be withdrawn due to its vulnerability to certain attacks [45]. Therefore, it should not be used as a cryptographic module in security systems, and, despite similar results evaluating the level of randomness at a level similar to AES, we strongly agree that the use of the 3DES algorithm as a core in CSPRBG should be avoided. In contrast, in the case of the 3DES and Twofish algorithms (which are known to be cryptographically weaker algorithms compared to AES [21]), the configuration of entropy sources is reflected in the NIST results by weakening or strengthening the level of randomness of the sequence generated by the prototype.

The bitstream generated by the wearable CSPRBG with the Twofish algorithm for entropy source configurations e4, e5, e6 shows truly random features. Increasing the number of entropy sources allows for an improvement in the random characteristics for the Twofish algorithm. However, increasing the number of entropy sources does not bring a significant improvement in the random characteristics for the 3DES algorithm, where there is a degradation in the NIST test results for the e4 and e6 cases.

In addition to the analysis with NIST tests, we performed a randomness study using the ENT tool, which uses a different set of tests to assess randomness as described in [46]. The use of the ENT tool, in some cases, can be treated as a preliminary randomness assessment for bitstreams created from experimental observation of physical phenomena [47]. However, for more rigorous knowledge of whether a particular sequence returned by a generator is random, one should use NIST tools that examine much longer bitstreams. Although entropy sources are the source of randomness, in the context of random generators it is more crucial to examine their statistical properties instead of Shannon entropy tests as is the case with the ENT tool. Therefore, NIST testing is the mandatory and most reliable procedure for assessing whether a random generator as a whole ensures the delivery of a random sequence [43].

The results in Table 5 show that for a low variation of entropy sources (e1–e3), the $\chi^{2}$ results indicate an entropy problem with the Twofish algorithm. In this test, significantly different values were obtained from those described in the manual for ENT [46]. In addition, for the e3 source, data compression is possible, which indicates repeated data patterns in the tested bitstream. For the 3DES algorithm, the ENT program indicates problems with the entropy sources e1, e4, e5 also in terms of the $\chi^{2}$ test.

## 6. Future Development of Wearable CSPRBG Platform

The wearable CSPRBG prototype presented in this paper has wired connections between the sensors and the trainer board with the FPGA. In future project development, these connections can be replaced by wireless communication, making the vest more functional and convenient to use. This communication will take place within the prototype itself without Internet access—ensuring that the benefit described in Section 1.1 is maintained. For securing wireless transmission, adaptation of some mechanisms from the Root of Trust concept can be considered. In addition, the use of wireless communication will allow the sensors to be mounted in various locations—including on the user’s body, e.g., a motion sensor mounted on a band worn on the wrist of the hand. Because of the prototype nature of the project, the textile components such as the vest and electronics used are relatively large compared to commercial wearable technology solutions. It should be noted that each system is first designed from a larger prototype and only its further development or even commercialization results in a significant miniaturization. It cannot be excluded that the sound data and the acceleration data may have specific output patterns according to the context. The purpose of this paper is not to study the impact of such attacks on the level of randomness obtained. However, the presented platform in the future can be used to study such attacks (typically taking place in the target environment), which aim to change the environmental conditions (such as movement and sound) monitored by sensors used in the presented wearable CSPRBG prototype.

**Figure 4:**
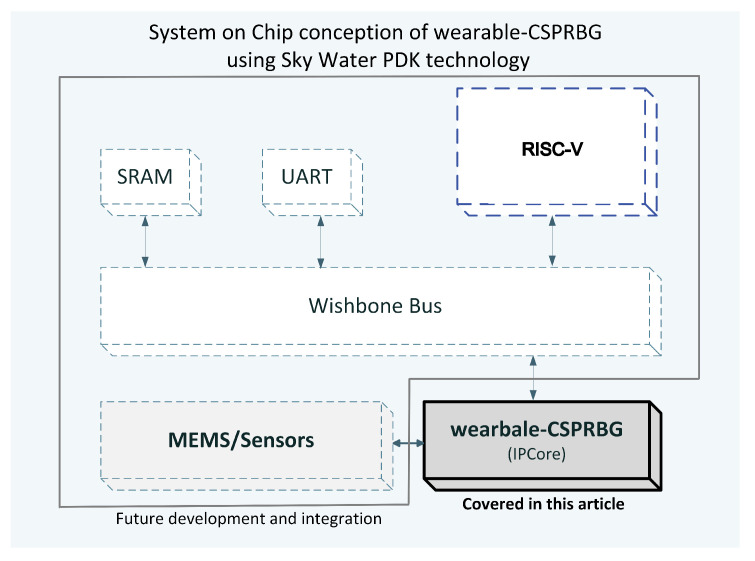
A potential approach to integrate the wearable CSPRBG as a SoC component module with an embedded RISC-V. Communication of all modules is via an open source hardware computer bus (Wishbone Bus), which allows the wearable CSPRBG to communicate with the RISC-V. For full functionality, the RISC-V module requires the addition of (a) Static Random-Access Memory (SRAM) for Linux or Read-Only Memory (ROM) storage, (b) a UART module for communication with other peripherals. Sensor fabrication in dedicated independent technologies was assumed.

In the farthest development horizon of the project, we plan to integrate the used sensors together with IPCore for Reduced Instruction Set Computer-five (RISC-V) as a dedicated seed generator function, Figure 4. Integration in such an approach will allow the created seed generator to be used in applications that use randomness and are run on the Linux operating system. The prototype with RISC-V chip can be taken as a starting point for further miniaturization of the wearable CSPRBG project. As part of the further development of this project, it is planned to work on the integration of sensors and the entire electronic layer as a System on Chip (SoC) using the SkyWater Process Design Kit (PDK) technology process [48] and the Caravel chip/template [49], which is a ready and tested implementation of the RISC-V processor prepared for the above-mentioned technology process. Conversion from hardware description language to the transistor level will be possible through the use of tools such as OpenLane [50,51,52]. Thanks to the use of Caravel, the prepared chip will allow, among other things, the undertaking of security tests related to attacks on the integrated entropy source in the context of hardware security and trust.

In our prototype, entropy was obtained from measurements made with external sensors. However, there are different approaches in the implementation of random generators as high integration scale electronic circuits, in which both a pseudo-random algorithm and entropy sources based on jitter and metastability are implemented within a single integrated circuit as described among others in [53]. If one assumes that the implementation of such solutions is done with ready IPCors, the process of simulation design and testing is relatively fast. It does not require the use of external modules responsible for aggregating entropy from the external environment. However, it should be noted that the approach of designing entropy sources based on the internal metastability of the chip should take into account the temperature changes at which the chip will ultimately generate random values [54]. Another important issue for all DRBGs is the threat from hardware Trojans implemented at the design stage of integrated electronics. The user must be sure that the entropy sources have not been deliberately designed to deliver values that are fixed and known by the attacker [17,20,55]. The approach proposed in wearable CSPRBG is a partial response to such threats. Another option for the development of our prototype is to be able to change its structure so that users can mix the entropy or directly the random sequence coming from the wearable CSPRBG with other external randomness generators—such as the one described in [53]. Such functionality would be consistent with the recommendations for DRBG design described in [9]. In such a concept, entropy and randomness would be generated ahead of two independent but interconnected electronic ecosystems. This combination would make attacks using hardware Trojans more difficult and would also eliminate potential problems if the ambient temperature affected the stability of the internal entropy source based on, for example, metastability. It is assumed that both electronic ecosystems could be integrated with the solution in Figure 4. In the FPGA approach, both solutions are comparable in terms of the cost of purchasing the necessary electronics. Thus, their integration should not generate additional costs.

One more direction of development of the prototype presented in the article could be to expand it by introducing additional facilities to guarantee increased randomness. It is worth noting here the potential of using neural networks as additional independent sources of entropy or as mechanisms to help increase the entropy available in the system [56,57,58,59,60].

## 7. Conclusions

The aim of this paper was to propose a modular cryptographically secure random number generator suitable for wearable technology. In almost every cryptographic system, the most difficult task is to design and directly implement a secure source of randomness. The specifics of wearable technology often exclude the integration of commercially available randomness generators due to their size and/or price. Therefore, it is necessary to look for new solutions in the way of randomness generation, suitable for the specifics of the application use. The prototype described in this paper has been implemented modularly which allows its migration to other hardware platforms based on FPGAs. The wearable CSPRBG structure allows one to increase the number of entropy sources in the future by adding new sensors measuring physical quantities such as temperature, illumination, humidity, atmospheric pressure, etc. The proposed prototype solution is a research contribution to the issues of limited access to EaaS services, as well as methods of self-designing systems free from the threat of introducing hardware Trojans by subcontractors or third-party IPCore providers.

The randomness analysis of the designed wearable CSPRBG showed that the AES algorithm is best suited for generating random bits. In the case of the Twofish algorithm, randomness is achieved if values from the chaotic generator, ciphertext loopback and plaintext are used in addition to the sensors to form the seed values. The vest wearable CSPRBG described in this paper is the platform for future research oriented towards the ultimate design of a cryptographic system completely independent of solutions offered by third-party providers.

## Figures and Tables

**Table 1:** IPCore used in the synthesis of wearable CSPRBG.

	License	Repository	Reference
AES	GPL	opencores.org	[34]
3DES	CoreTex Systems	opencores.org	[35]
TWOFISH	GPL	opencores.org	[36]
PmodMICRefComp	Digilent Ro.	reference.digilentinc.com	[37]
SPI	LGPL	opencores.org	[38]
SD	Steven J. Merrifield	stevenmerrifield.com	[39]
SHA-3	Team Keccak	keccak.team	[40]

**Table 2:** FPGA Hardware Switch Configurations in Figure 2.

Symbol on Figure 2	Tag on ZYBO Device	Function
sw(0)	SW0	System reset;
		active with high state
sw(1)	SW1	System start-up;
		active with high state
sw(2)	SW2	not connected
sw(3)	SW3	enable/disable the
		random data recording

**Table 3:** Comparison of FPGA hardware resources used for the AES, 3DES and TWOFISH algorithms.

	# of Resources Used			Utilization Percent			
FPGA Resource *	AES	3DES	TWOFISH	AES	3DES	TWOFISH	Max
(# Available)							diff
LUT (17600)	7576	7594	8554	43.0%	43.1%	48.6%	5.6%
LUTRAM (6000)	66	192	66	1.1%	3.2%	1.1%	2.1%
FF (35200)	5511	5632	5621	15.7%	16.0%	16.0%	0.3%
BRAM (60)	11	2	6	18.3%	3.3%	10.0%	15.0%
DSP (80)	48	48	48	60.0%	60.0%	60.0%	0.0%
IO (100)	19	19	19	19.0%	19.0%	19.0%	0.0%
BUFG (32)	5	5	6	15.6%	15.6%	18.8%	3.1%
MMCM (2)	1	1	1	50.0%	50.0%	50.0%	0.0%

* see Abbreviations section for acronym details.

**Table 4:** NIST test results for wearable CSPRBG with varying entropy source configurations shown in Figure 3.

		Test Name															
Algorithm Name	Entropy Source	Frequency	Ferequency within a Block	Runs	Longest Run of Ones	Rank	Spectral	Non- Overlapping	Overlapping	Maurer’s Universal	Serial	Approximate Entropy	Cumulative Sums	Random Excursions	Random Excursions Variant	Linear Complexity	Failed Tests
**AES**	e1	0	0	0	0	0	0	1/148	0	0	0/2	0	0/2	0/8	0/18	0	1/188
	e2	0	0	0	0	0	0	1/148	0	0	0/2	0	0/2	0/8	0/18	0	1/188
	e3	0	0	0	0	0	0	1/148	0	0	0/2	0	0/2	1/8	0/18	0	2/188
	e4	0	0	0	0	0	0	0/148	0	0	0/2	0	0/2	0/8	1/18	0	1/188
	e5	0	0	0	0	0	0	0/148	0	0	0/2	0	0/2	0/8	0/18	0	0/188
	e6	0	0	0	0	0	0	0/148	0	0	0/2	0	0/2	0/8	0/18	0	0/188
**3DES**	e1	0	1	0	0	1	0	0/148	0	1	2/2	1	0/2	0/8	0/18	0	5/188
	e2	0	1	0	0	0	0	1/148	0	1	2/2	1	0/2	0/8	0/18	0	4/188
	e3	-	-	-	-	-	-	-	-	-	-	-	-	-	-	-	-
	e4	0	1	0	0	1	1	3/148	0	1	0/2	1	0/2	0/8	0/18	0	8/188
	e5	0	1	0	0	1	0	1/148	0	1	0/2	1	0/2	0/8	0/18	0	5/188
	e6	1	1	0	0	1	0	5/148	0	1	2/2	1	1/2	0/8	0/18	0	11/188
**TWOFISH**	e1	1	0	0	0	0	0	20/148	0	0	1/2	1	2/2	0/8	0/18	0	25/188
	e2	1	1	1	0	0	0	36/148	0	0	1/2	1	2/2	0/8	0/18	0	43/188
	e3	-	-	-	-	-	-	-	-	-	-	-	-	-	-	-	-
	e4	0	0	0	0	0	0	0/148	0	0	0/2	0	0/2	0/8	0/18	0	0/188
	e5	0	0	0	0	0	0	0/148	0	0	0/2	0	0/2	0/8	0/18	0	0/188
	e6	0	0	0	0	0	0	0/148	0	0	0/2	0	0/2	0/8	0/18	0	0/188

**Table 5:** Results for randomness evaluation for w-CSPRBG for varying entropy sources obtained from ENT suits tests, where Comp is the Compression ratio, AMV is the Arithmetic Mean Value, MC is the Monte Carlo value for π and SCC is the Serial Correlation Coefficient.

Algorithm Name	Entropy Sources	Entropy	Comp %	$\chi^{2}$; %	AMV	MC π %	SCC
**AES**	e1	7.999999	0	215.84; 90	127.5081	0	0.000169
	e2	7.999999	0	219.26; 90	127.5009	0.01	−0.000011
	e3	7.999999	0	273.84; 25	127.5055	0.01	0.00001
	e4	7.999999	0	248.23; 50	127.508	0.01	−0.000118
	e5	7.999999	0	256.58; 50	127.5026	0.02	0.000004
	e6	7.999999	0	254.27; 50	127.5068	0.01	0.000179
**3DES**	e1	7.999999	0	371.06; 0.01	127.5076	0.13	0.015556
	e2	7.999998	0	285.66; 10	127.505	0.12	0.015738
	e3	-	-	-	-	-	-
	e4	7.999998	0	312.24; 1	127.5025	0.11	0.015595
	e5	7.999998	0	296.90; 5	127.5068	0.11	0.015703
	e6	7.999998	0	281.63; 25	127.4937	0.12	0.015712
**Twofish**	e1	7.998618	0	256004.59; 0.01	127.7015	0.23	0.000408
	e2	7.999877	0	21359.31; 0.01	126.7808	0.34	−0.018447
	e3	6.598928	17	554932457.50; 0.01	124.6524	7.93	−0.098951
	e4	7.999999	0	225.75; 90	127.4936	0	−0.000138
	e5	7.999999	0	259.01; 50	127.5029	0.01	−0.00013
	e6	7.999999	0	239.56; 50	127.5027	0	−0.000148

## Data Availability

Data are contained within the article.

## Abbreviations

The following abbreviations are used in this manuscript:

AES	Advanced Encryption Standard
AMV	Arithmetic Mean Value
ASIC	Application Specific Integrated Circuit
BRAM	Block Random Access Memory
BUFG	Global clock buffer
Comp	Compression Ratio
CSPRBG	Cryptographically Secure Pseudo-Random Bit Generator
DRBG	Deterministic Random Bit Generator
DRNG	Deterministic Random Number Generators
DSP	Digital Signal Processing
EaaS	Entropy as a Service
FF	Flip Flop
FPGA	Field Programmable Gate Array
GPU	Graphics Processing Unit
IO	Input Output
IoT	Internet of Things
LUT	LookUp Table
LUTRAM	LookUp Table Random Access Memory
NIST	National Institute of Standards and Technology
MC	Monte Carlo Value for π
MMCM	Mixed-Mode Clock Manager
PDK	Process Design Kit
PRBG	Pseudo-Random Bit Generator
RAM	Random Access Memory
RISC	Reduced Instruction Set Computer
RISC-V	Reduced Instruction Set Computer-five
ROM	Read-Only Memory
SCC	Serial Correlation Coefficient
SHA	Secure Hash Algorithm
SoC	System on Chip
SPI	Serial Peripheral Interface
SRAM	Static Random-Access Memory
TRBG	True Random Bit Generator
UART	Universal Asynchronous Receiver-Transmitter
USB	Universal Serial Bus
VHDL	Very High-Speed Integrated Circuit Hardware Description Language
VHDL-AMS	VHDL for Analog and Mixed Signals
ZYBO	ZYnq BOard
